# Asymmetric Sulfoxidation by a Tyrosinase Biomimetic Dicopper Complex with a Benzimidazolyl Derivative of L-Phenylalanine

**DOI:** 10.3390/molecules28114487

**Published:** 2023-06-01

**Authors:** Eliana Lo Presti, Fabio Schifano, Chiara Bacchella, Laura Santagostini, Luigi Casella, Enrico Monzani

**Affiliations:** 1 Dipartimento di Chimica, Università di Pavia, Via Taramelli 12, 27100 Pavia, Italy; eliana.lopresti01@gmail.com (E.L.P.); fabio.schifano01@universitadipavia.it (F.S.); chiara.bacchella@unipv.it (C.B.); luigi.casella@unipv.it (L.C.); 2 Dipartimento di Chimica, Università di Milano, Via Golgi 19, 20133 Milano, Italy; laura.santagostini@unimi.it

**Keywords:** copper, dioxygen, sulfoxidation, stereoselective catalysis, kinetics

## Abstract

A challenge in mimicking tyrosinase activity using model compounds is to reproduce its enantioselectivity. Good enantioselection requires rigidity and a chiral center close to the active site. In this study, the synthesis of a new chiral copper complex, [Cu_2_(mXPhI)]^4+/2+^, based on an *m*-xylyl-bis(imidazole)-bis(benzimidazole) ligand containing a stereocenter with a benzyl residue directly bound on the copper chelating ring, is reported. Binding experiments show that the cooperation between the two metal centers is weak, probably due to steric hindrance given by the benzyl group. The dicopper(II) complex [Cu_2_(mXPhI)]^4+^ has catalytic activity in the oxidations of enantiomeric couples of chiral catechols, with an excellent discrimination capability for Dopa-OMe enantiomers and a different substrate dependence, hyperbolic or with substrate inhibition, for the L- or D- enantiomers, respectively. [Cu_2_(mXPhI)]^4+^ is active in a tyrosinase-like sulfoxidation of organic sulfides. The monooxygenase reaction requires a reducing co-substrate (NH_2_OH) and yields sulfoxide with significant enantiomeric excess (e.e.). Experiments with ^18^O_2_ and thioanisole yielded sulfoxide with 77% incorporation of ^18^O, indicating a reaction occurring mostly through direct oxygen transfer from the copper active intermediate to the sulfide. This mechanism and the presence of the chiral center of the ligand in the immediate copper coordination sphere are responsible for the good enantioselectivity observed.

## 1. Introduction

**Chart 1:**
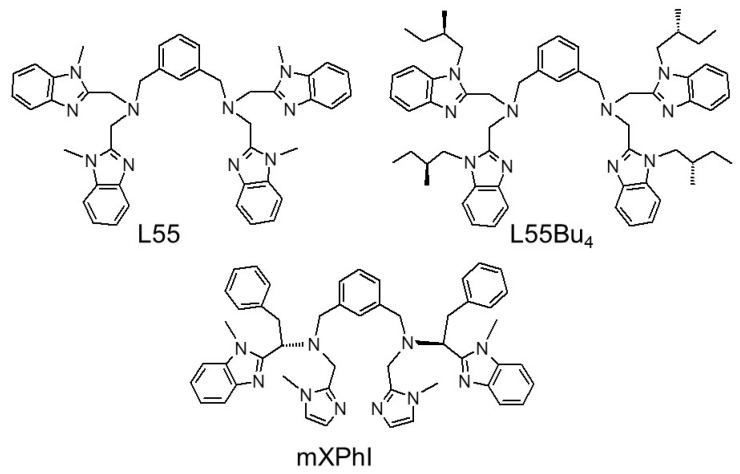
Structure of ligands L55, L55Bu4, and mXPhI.

The activities of the copper enzyme tyrosinase, the structure of which in the met form is shown in Figure S1, have been the subject of several studies using model systems [1,2,3,4]. In the framework of tyrosinase biomimetic systems, the dinuclear copper(II) complex of the bis(amino)-*m*-xylyl-tetrabenzimidazole ligand L55 [5,6,7,8] is considered one of the most efficient biomimetic complexes for catecholase activity [9]. The aim of progressing toward biomimetic systems that can promote stereoselective oxidations has prompted suitable modifications of the ligand skeleton, generating the chiral L55 analogue L55Bu_4_, carrying the chiral moieties on the substituents of the benzimidazole groups (Chart 1) [10]. The corresponding dinuclear copper(II) complex displays moderate efficiency in catalyzing enantio-discriminating catecholase activity, except for the pair of L/D-Dopa methyl ester substrates, for which the discrimination was high, in favor of the L-enantiomer [10]. Regarding other types of stereoselective oxidations, complex [Cu_2_(L55Bu_4_)]^4+^ was assayed in the catalytic sulfoxidation of thioanisole, but the product was obtained with a modest 12% e.e. [10]. This result was explained in terms of the large separation of the ligand stereocenters from the dicopper catalytic center, as the chiral alkyl substituents of the four benzimidazoles are oriented outside the cavity where the metal ions, dioxygen, and sulfide react with each other [9]. Other chiral dinuclear complexes with ligands bearing imidazoles replacing the benzimidazole groups were also reported (EHI [11] and mXHI [12]), and their enantio-discriminating oxidations were studied. Complex [Cu_2_(mXHI)]^4+^ exhibited good enantio-discriminating capacity towards chiral, polar catechols but poor enantioselective properties in the asymmetric sulfoxidation of hydrophobic sulfides [12].

**Scheme 1:**
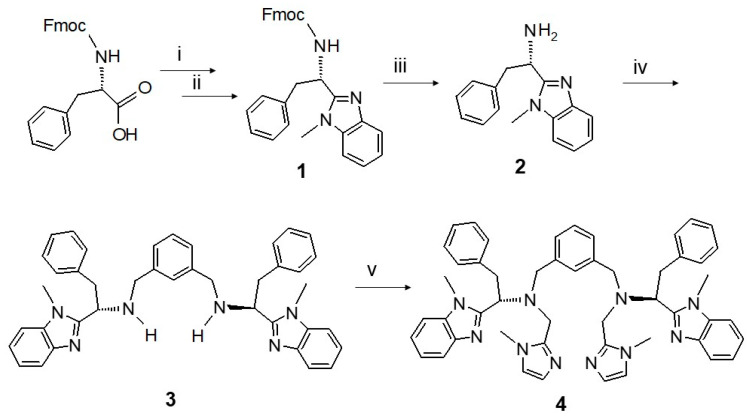
The multistep synthesis of ligand mXPhI by functionalization of L-phenylalanine. (i) *N*-methyl *ortho*-phenylenediamine, HBTU (2-(1H-benzotriazol-1-yl)-1,1,3,3-tetramethyluronium hexafluorophosphate), acetonitrile, r.t., overnight; (ii) glacial acetic acid, 70 °C, 4 h; (iii) dichloromethane/piperidine 20% (*v*/*v*), r.t, 30 min; (iv) isophthaldialdehyde, dichloromethane, sodium triacetoxy borohydride; (v) 1-methyl-2-carbomethoxy imidazole, dichloroethane, sodium triacetoxy borohydride.

Based on the idea that bringing the stereocenters closer to the dicopper-substrate reaction site will increase the chiral information that can be transferred, we have developed a new chiral ligand in which the donor benzimidazole groups are built in the structure of L-phenylalanine. To achieve the biomimetic 3N donor set at each copper center, further derivatization with imidazole donors of the ligand has been carried out, yielding the ligand labeled mXPhI (Scheme 1). This ligand can be considered as the prototype of a family of chiral bis(amino)-bis(imidazole)-bis(benzimidazole) ligands that, in principle, could be derived from any α-amino acid.

The dinuclear complex [Cu_2_(mXPhI)]^4+^ is thus inspired by [Cu_2_(L55)]^4+^, in which the catalytic efficiency depends on the presence of five-membered chelate rings in the coordination sphere of each copper(II) center. The X-ray structures of both the dicopper(II) and dicopper(I) complexes of L55 are known [6], and we assume a similar coordination set for the parent [Cu_2_(mXPhI)]^4+/2+^ complexes, given that the ligand provides a similar system of fused chelate rings. In the newly conceived strategy for the ligand mXPhI, a benzimidazole ring is derived from functionalization of the carboxylic group of a chiral α-amino acid, allowing the stereocenter to be placed within the chelate ring and thus in closer proximity to the metal center. The choice of L-phenylalanine allows the aromatic side chain to be placed in the second sphere around the metal ions, where it could possibly stabilize the binding of hydrophobic ligands or substrates to the metal center. It is apparent that such ligand design could be easily extended to incorporate into the ligand other amino acid moieties, making it possible to vary the nature of the residues and side chains in the immediate environment of the catalytic metal centers.

Herein, we describe the synthesis of the chiral dinuclear complex [Cu_2_(mXPhI)]^4+^, the cooperation of its two metal ions in the binding of anions, the enantio-discriminating catecholase activity toward chiral biologically relevant catechols, and the monooxygenase catalytic activity in the sulfoxidation of aromatic sulfides in the presence of reducing co-substrates. Experiments with ^18^O_2_ for the last-mentioned reactivity showed the presence of two reaction mechanisms, a direct oxygen transfer, leading to good enantiomeric excess, and a radical process leading to racemic sulfoxides.

## 2. Results and Discussion

### 2.1. Synthesis and Characterization

The synthesis of mXPhI reported in Scheme 1 starts from *N*-Fmoc-protected L-phenylalanine, which was transformed into the α-amino-*N*-methyl benzimidazole derivative **1** in a two-step reaction involving a coupling between the carboxylic portion of the amino acid and *N*-methyl-*ortho*-phenylenediamine, followed by ring closure with elimination of water, promoted by glacial acetic acid [13]. Fmoc-deprotection of compound **1**, in dichloromethane with 20% (*v*/*v*) of piperidine, gave **2**, in quantitative yield. Two of these chiral α-amino benzimidazole compounds were connected by stepwise reductive amination with isophthaldialdehyde, giving compound **3,** which, after a subsequent reductive amination with 1-methyl-2-imidazole carboxaldehyde, gave ligand **4** (mXPhI). The dinuclear copper(II) complex of mXPhI was obtained by reaction of the ligand with copper(II) salt. 

The electronic spectrum of [Cu_2_(mXPhI)]^4+^ in the UV region is dominated by the high intensity absorption band of the benzimidazole chromophores of the ligand around 290 nm (see Figure S2 in Supplementary Materials). On the low-energy tail of this band, in the range between 300 and 350 nm, the typically featureless absorptions due to π(benzimidazole) and π(imidazole) to Cu^II^ LMCT observed for this family of complexes can be noted [5,6,10,11,12]. These LMCT features will be more clearly observed in the CD spectrum of [Cu_2_(mXPhI)]^4+^ to be discussed below. The visible region is dominated by a nonsymmetrical absorption band centered at 710 nm, with a prominent shoulder near 900 nm, attributable to d-d metal transitions (Figure S2). The pattern of these d-d bands is clearly suggestive of a trigonal bipyramidal coordination geometry for the copper(II) centers [14]. 

**Figure 1:**
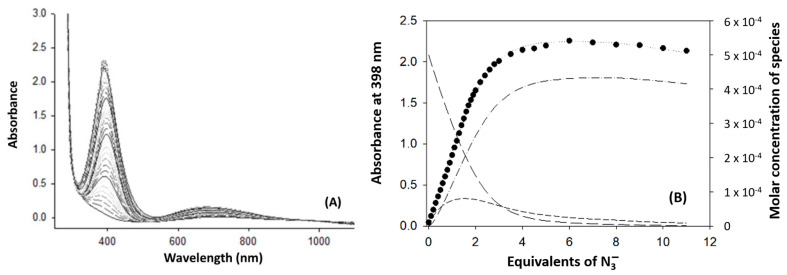
(**A**) Family of UV-vis spectra taken upon addition of a concentrated solution of NaN_3_ (4 × 10^−2^ M) to [Cu_2_(mXPhI)]^4+^ in methanol solution (5 × 10^−4^ M). (**B**) Distribution diagram (concentration vs. equivalent of N_3_^−^) of the species. Long and short dashed lines: free [Cu_2_(mXPhI)]^4+^ and [Cu_2_(mXPhI)(N_3_)]^3+^, respectively; dashed–dotted line: [Cu_2_(mXPhI)(N_3_)_2_]^2+^. The graph shows the experimental profile of absorbance vs. equiv. NaN_3_ at 398 nm (black circles) and the fitted curve (dotted line).

**Figure 2:**
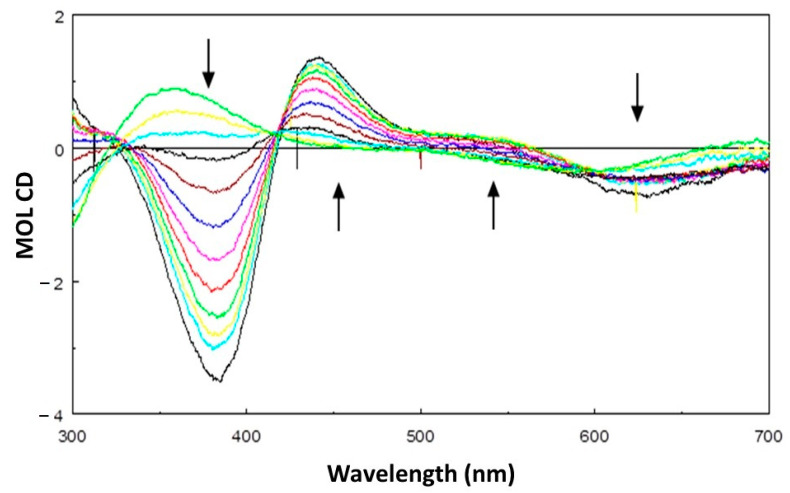
CD spectra upon titration of [Cu_2_(mXPhI)]^4+^ with 0, 0.2, 0.4, 0.6, 0.8, 1.0, 1.2, 1.4, 1.6, 1.8, 2.0, and 5.0 azide equiv. in methanol solution. The arrows indicate the order of development of the bands with the addition of azide.

**Chart 2:**
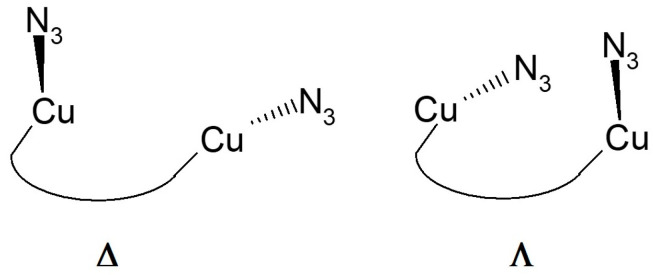
Chirality of the two possible skew arrangements of Cu-azido bonds in the Cu-azide adducts of dinuclear copper(II) complexes [19].

We have systematically employed binding experiments with the azido ligand to assess the accessibility and mode of binding (terminal vs. bridging) of small molecules to the dicopper(II) centers of biomimetic complexes [10,11,12,15]. The addition of azide to a solution of [Cu_2_(mXPhI)]^4+^ in methanol produces the growth of the characteristic near-UV band at about 400 nm, attributable to π(azido)→Cu(II) LMCT (Figure 1A) [15,16,17]. In the present case, the LMCT grows in with a symmetric shape, with only a slight red shift with the progress of the titration, which suggests that the ligand binds in the terminal mode to each of the two copper(II) centers. However, the CD spectra recorded at various stages of the titration clearly show a splitting of the azido→Cu(II) LMCT into two components, with a negative extremum at 380 nm and a positive peak near 440 nm (Figure 2). We interpret this splitting of the CD LMCT bands as due to exciton coupling of the two transition moments of the Cu-azido chromophores [18], as we have previously observed for dicopper(II) complexes with the chiral ligands PHI and mXHI [19]. However, the CD pattern in the present case is the opposite, with a negative CD extremum at higher energy and a positive component at lower energy, indicating that the chirality of the skew arrangement of the Cu-N(azido) linkages is opposite, and hence Λ (Chart 2). The azide binding behavior of [Cu_2_(mXPhI)]^4+^ is thus different from that of the parent [Cu_2_(L55)]^4+^ [15] and [Cu_2_(L55Bu_4_)]^4+^ [10] complexes, where azido ligands bridge in μ-1,1 mode between the copper(II) centers. This is probably motivated by steric reasons, due to the substituents on the donor groups that may prevent a closer approach between the two copper(II) centers. The tendency of [Cu_2_(mXPhI)]^4+^ to work as a mononuclear complex is also shown by its catecholase-like activity; see below.

Also in the visible range, the optical activity of the [Cu_2_(mXPhI)]^4+^ complex and its azido adducts is significant, unlike what was observed previously for the parent [Cu_2_(L55Bu_4_)]^4+^ derivative [10]. This is due to the fact that in [Cu_2_(mXPhI)]^4+^ the chiral centers are within the amino acid chelate ring, and the dominant negative sign of the CD d-d bands suggests the predominance of a λ conformation [19]. Unlike what was observed for the histidine-derived complex [Cu_2_(PHI)]^4+^ [11], in this case the addition of azide does not cause an inversion of the amino acid chelate ring, with inversion of the CD sign, indicating that the bound azido ligand can be accommodated in an accessible equatorial coordination position. 

Together with the changes in the near-UV range, during the titration with azide it is possible to note a marked progressive blue shift and a hyperchromic effect of the d-d band, to about 630 nm. The increase in intensity is due to the coupling of d-d transitions with the azido→Cu(II) LMCTs, whereas the blue shift reflects a change in the stereochemistry of the copper(II) centers toward a square pyramidal arrangement [14]. By analyzing the titration data at 398 nm it was possible to extrapolate two binding equilibria, related to the following equilibria:

[Cu_2_(mXPhI)]^4+^ + N_3_^−^ ⇌ [Cu_2_(mXPhI)(N_3_)]^3+^

[Cu_2_(mXPhI)(N_3_)]^3+^ + N_3_^−^ ⇌ [Cu_2_(mXPhI)(N_3_)_2_]^2+^

with two close binding constants and logβ_2_ = 7.07 [15,19].

**Figure 3:**
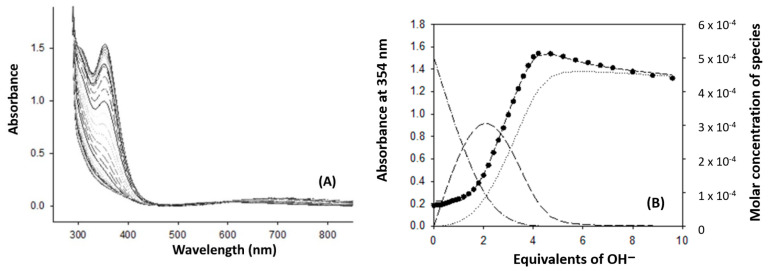
(**A**) UV-vis spectra recorded upon titration of [Cu_2_(mXPhI)]^4+^ (5 × 10^−4^ M) with sodium hydroxide (4 × 10^−2^ M) in methanol. (**B**) Species distribution diagram; the plots show the experimental profile of absorbance vs. equiv. NaOH at 354 nm (black circles) and the fitted curve (dashed line); the dashed–dotted line corresponds to [Cu_2_(mXPhI)]^4+^ concentration, the medium dashed line to [Cu_2_(mXPhI)(OH)_2_(S)_2_]^2+^ species (with S = methanol or water), and the dotted line to the [Cu_2_(mXPhI)(µ-OH)_2_]^2+^, or [Cu_2_(mXPhI)(µ-OH)_2_(OH)_2_], species.

**Scheme 2:**
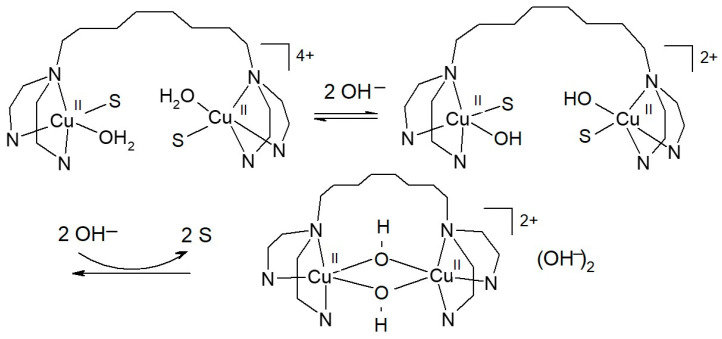
Pathway of [Cu_2_(mXPhI)(H_2_O)_2_]^4+^ conversion to the di-hydroxo-bridged [Cu_2_(mXPhI)(µ-OH)_2_]^2+^ species. As in solution water molecules are exchangeable with solvent, we have introduced solvent molecules (S) in the Cu coordination sphere, but leaving the coordinated water that gives rise to the various Cu-hydroxo species evidenced by UV-vis and CD signatures.

Similarly to [Cu_2_(L55)]^4+^ [15] and [Cu_2_(L55Bu_4_)]^4+^ [10], complex [Cu_2_(mXPhI)]^4+^ exhibits pH-dependent features in the electronic spectra. Changes in the spectrum observed upon titration with sodium hydroxide consist of the development of a prominent near-UV absorption band at 354 nm, accompanied by a shoulder near 310 nm, on the tail of a higher energy absorption (Figure 3). These bands are due to coupled hydroxo→Cu(II) LMCT transitions. The addition of hydroxide also produced a marked blue shift of the d-d bands from 710 nm to about 600 nm, indicating a transition from trigonal bipyramidal to square pyramidal coordination geometry [14]. It is interesting to note that deprotonation of coordinated water in the dinuclear complex does not proceed stepwise. From 0 to 2 equivalents of added hydroxide, only a weak absorption increase near 350 nm was in fact observed, whereas more significant changes in the near-UV range took place progressively with the addition of further hydroxide equivalents, from 2 equivalents to 4 equivalents. This indicates that a double hydroxo-bridged complex is formed in a single step (Scheme 2). 

Analysis of experimental data shows that three species are involved in the equilibrium. Deprotonation of the first two water molecules leaves the complex in the trigonal bipyramidal structure, with one water and one OH^−^ group coordinated in terminal mode. Further addition of hydroxide leads to the bis-µ-hydroxo species inducing a transition from trigonal bipyramidal to square pyramidal or eventually distorted octahedral structure, with weakly bound water, at copper(II) centers (Scheme 2). 

### 2.2. Enantioselective Oxidation of Sulfides

The dinuclear complex [Cu_2_(L55)]^4+^ was found to catalyze the monooxygenation of thioanisole and other sulfides in the presence of hydroxylamine as the auxiliary reducing agent [20]. A main reason for developing chiral analogues such as [Cu_2_(mXPhI)]^4+^ was the attempt to induce enantioselectivity in the monooxygenase reaction at sulfur. The reaction has precedent because it is catalyzed by tyrosinase [21]. The enzyme is efficient and gives sulfoxide with excellent yield and enantiomeric excess (e.e.) but exhibits restrictions in the type of sulfides that can be oxidized, probably due to limitations in access to the dicopper active site. In addition, organic sulfides are typically hydrophobic molecules with low solubility in an aqueous medium. For biomimetic compounds, these limitations can be overcome, as substrate accessibility to the dicopper center is much higher, and they can work in aqueous–organic solvent; therefore, they represent a promising alternative. We studied the sulfoxidation of two representative methyl aryl sulfides in methanol–acetate buffer (50 mM, pH 5.1) 9:1 solution, using a high ratio of 1000:1 between substrate and [Cu_2_(mXPhI)]^4+^, and varying the concentration of hydroxylamine, to find the best conditions, in terms of yield and e.e. of sulfoxide. The results are collected in Table 1. The product sulfoxides were formed with a preference for the (S) enantiomer.

**Scheme 3:**
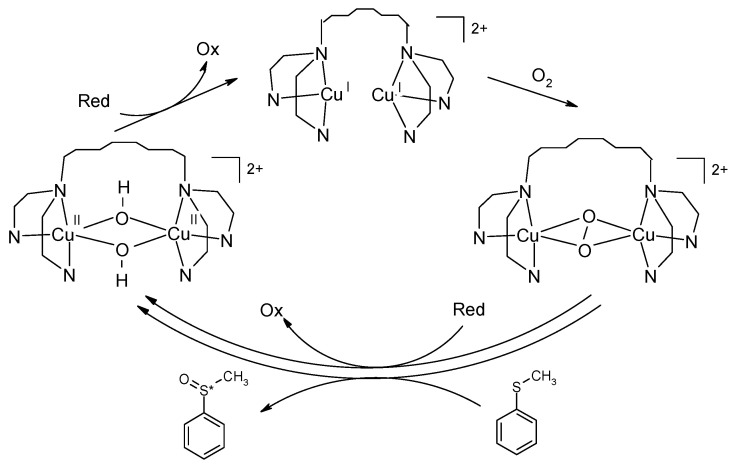
Simplified mechanism of the catalytic cycle of the sulfoxidation reaction: “Red” and “Ox” represent the reduced and oxidized forms, respectively, of the auxiliary reductant, hydroxylamine in the present case, and the copper-dioxygen intermediate is assumed in the η^2^:η^2^-peroxodicopper(II) form. * Indicates the chiral center of the sulfoxide.

For thioanisole, the trend shown in Figure S3 and Table 1 highlights how the reaction yield decreases exponentially when the concentration of reducing agent increases. The enantiomeric excess, instead, undergoes a rapid increase between 0 and 1 mM of hydroxylamine, followed by a marked decline. This behavior depends on the nature of the reducing agent, considering that there is competition between the oxidation of the sacrificial reductant and the substrate monooxygenation. As shown by the catalytic cycle represented in Scheme 3, the auxiliary reductant is necessary to recycle the copper catalyst from Cu^2+^ to Cu^+^ but can also compete with the sulfide substrate in the oxygen transfer step involving the copper-dioxygen intermediate, assumed here to be in η^2^:η^2^-peroxodicopper(II) form, as in tyrosinase [22,23]. Hydroxylamine is practically useful as an auxiliary reagent but bears a complex redox chemistry that can generate radicals. Therefore, on increasing hydroxylamine concentration, a radical pathway of sulfur oxidation assumes a prominent role, as one-electron oxidation prevails on the oxygen transfer from the Cu_2_O_2_ species, thus making the influence of the chiral catalyst negligible. The catalyst itself undergoes inactivation with time by these radical reactions. It is noteworthy, though, that even if sulfoxide yields are limited, the reaction is still catalytic, as the catalyst makes several turnover cycles before inactivation. 

When methyl p-tolyl sulfide was used as substrate, low yields were obtained in all experiments, but the reaction still gives useful information (Table 1 and Figure S4). Yields of sulfoxide were lower than for thioanisole, but the maximum enantiomeric excess was similar. The low yield may be attributed to the poor ability of this bulky substrate to compete with the oxidation of hydroxylamine. An appreciable enantiomeric excess of about 40% was obtained at low concentrations of reductant; however, the e.e. steadily decreased with increased concentrations of hydroxylamine, and at equimolar [sulfide] and [hydroxylamine] the resulting product was racemic. This can be explained, considering that methyl p-tolyl sulfide is more electron rich than thioanisole, and its oxidation through a radical pathway will be easier.

Important mechanistic information can be obtained through labeling experiments with ^18^O_2_, since a high level of incorporation of labeled oxygen into the product sulfoxide can only be accounted for by the monooxygenase pathway (Scheme 3), where a ternary intermediate between Cu_2_/O_2_/sulfoxide can be formed. The sulfoxidation of thioanisole was replicated in an atmosphere of ^18^O_2_, operating in the best conditions (in terms of e.e) to reduce the impact of the competing radical reactions (1 mM of hydroxylamine). In these conditions, the catalytic oxidation of thioanisole by [Cu_2_(mXPhI)]^4+^ yielded sulfoxide with 77% incorporation of ^18^O (Figure S5). These results indicate that the reaction occurs with oxygen transfer from the Cu_2_O_2_ intermediate to the sulfide, with only a minor contribution by the radical pathway. 

### 2.3. Oxidation of Chiral Catechols: Enantio-Discriminating Catecholase Activity

**Figure 4:**
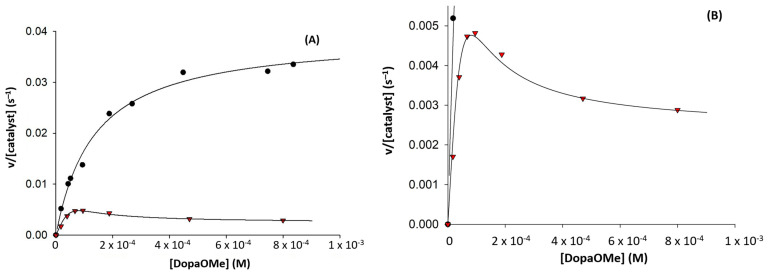
(**A**) Oxidation rate data at increasing substrate concentration for L-Dopa methyl ester (black circles), and D-Dopa methyl ester (red triangles). (**B**) Expansion of the plot for the D enantiomer.

The catechol oxidase activity of [Cu_2_(mXPhI)]^4+^ was also investigated for comparison with the parent complexes studied before. As with [Cu_2_(L55Bu_4_)]^4+^ [10], [Cu_2_(mXPhI)]^4+^ exhibits no significant discrimination for the enantiomeric substrates L/D-Dopa and R/S-norepinephrine (Table 2, Figure S6). Additionally, in this case, we ascribe the lack of chiral recognition for the two enantiomers of norepinephrine to the large distance between the stereogenic centers of the substrate and those of the ligand in the adduct preceding the electron transfer step, as they are too distant for significant interactions. Similar considerations can be proposed for the two enantiomers of Dopa, with the additional competitive effect exerted by the amino acid portion of the substrate toward the catechol moiety in the binding to the dicopper(II) complex. This is supported by the finding that when one of the chelating donors of the amino acid substrate is lost, chiral discrimination can be observed. Indeed, for L/D-Dopa methyl ester substrates (DopaOMe), an interesting difference in oxidation rate for the two enantiomers was found. While the rate data at increasing substrate concentration followed a typical Michaelis–Menten trend for the L-enantiomer, the D-enantiomer exhibited substrate inhibition (Figure 4).

**Scheme 4:**
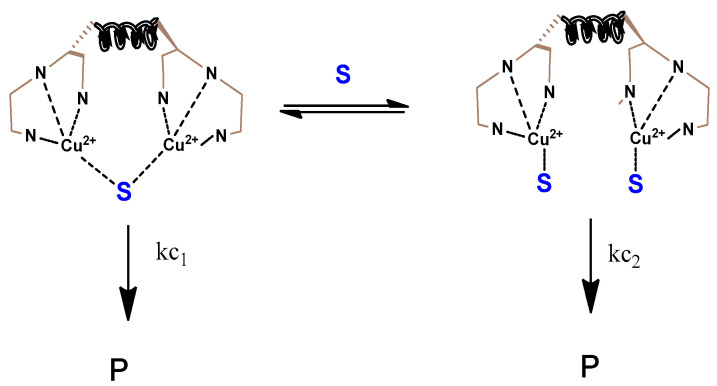
Schematic model representing the reactive intermediates involved in the competing pathways of oxidation of D-Dopa methyl ester; S is D-Dopa methyl ester, and P is the oxidation product.

As shown by the expanded plot in Figure 4, substrate inhibition at a high concentration of D-Dopa methyl ester is not complete, as the rate of oxidation did not drop to zero; this could be explained by assuming that, in the saturating condition, two molecules of substrate can be bound by the dicopper complex, and this form is less but still reactive (Scheme 4). The equation we used to fit the rate data is the following:

(1) $$\frac{v}{\left[catalyst\right]}=\frac{k_{c1}\left[S\right]+k_{c2}K_{B2}{\left[S\right]}^{2}}{K_{M}+\left[S\right]+K_{B2}{\left[S\right]}^{2}}$$

where *k*_*c*1_ and *k*_*c*2_ are the oxidation rates for the 1:1 and the 1:2 complex/substrate adducts, respectively, and *K*_*B*2_ is the dissociation constant for the bis-substrate adduct of the complex (see the Supporting Information for derivation of this equation). The model used to interpret the behavior of D-Dopa methyl ester is adequate, as the fit of the data is good (Figure 4), but it was impossible to extrapolate the four parameters due to the high intercorrelation of the experimental data.

## 3. Materials and Methods

All reagents and solvents from commercial sources were of the highest purity available and were used as received. Elemental analyses were obtained at the microanalysis service of the Milano CIMA department. CD spectra were obtained with a Jasco J500 spectropolarimeter (JASCO Corporation, Tokyo, Japan); spectra were recorded in the range 300–700 nm, at 50 nm/min, with three scans acquired for each spectrum, and 0.2 nm resolution. NMR spectra were recorded on a Bruker AVANCE 400 spectrometer (Bruker Italia, Milan, Italy), operating at 9.37 T. Data acquisition and processing were performed using a standard Bruker software package (Topspin 1.3). UV-vis spectra were recorded on an Agilent 8453 spectrophotometer. HPLC analyses were performed on a Jasco HPLC instrument (JASCO Corporation, Tokyo, Japan) equipped with two PU-1580 pumps and an MD-1510 diode array detector (working range: 195–659 nm) or on a Shimadzu HPLC instrument (Shimadzu Corporation, Kyoto, Japan) equipped with two LC-20AD pumps and an SPD-M20A diode array detector (working range: 190–800 nm). Mass spectra were recorded using a Thermo-Finnigan LCQ ADV MAX spectrometer (Thermo-Finnigan, San Jose, CA, USA).

### 3.1. Synthesis of (9H-fluoren-9-yl)methyl(1-(1-methyl-1H-benzoimidazol-2-yl)-2-phenylethyl)carbamate (***1***)

HBTU (2-(1H-benzotriazol-1-yl)-1,1,3,3-tetramethyluronium hexafluorophosphate) (0.173 g, 0.464 mmol) was dissolved in 5 mL acetonitrile at 0 °C, bubbling argon into the solution. Then, 0.150 g of *N*-Fmoc protected L-phenylalanine (0.387 mmol) was added to the solution, followed by 0.073 g of *N*-methyl *ortho*-phenylenediamine dihydrochloride (0.464 mmol) and 82.5 µL of diisopropylethylamine (0.464 mmol), continuing bubbling of argon for 15 min. The reaction mixture was allowed to reach room temperature, with stirring overnight. After rotary evaporation of the solvent, 5 mL of acetic acid was added to the solution, which was then heated to 70 °C for 4 h. The reaction was stopped by evaporating the acetic acid and directly purified by column chromatography with silica 230–400 mesh as stationary phase and hexane:ethyl acetate 6:4 mixture as eluent (R_f_: 0.4). The product obtained was 0.026 g (yield 14%). MS: + 474 (M+H^+^), ^1^H NMR (400 MHz, CDCl_3_): δ 7.79 (dd, *J* = 17.9, 7.4 Hz, 1H), 7.57 (dd, *J* = 13.8, 7.5 Hz, 1H), 7.40 (t, *J* = 7.3 Hz, 1H), 7.35–7.23 (m, 2H), 7.08 (d, *J* = 2.7 Hz, 1H), 6.48 (d, *J* = 8.4 Hz, 1H), 5.30 (t, *J* = 9.5 Hz, 1H), 4.38 (ddd, *J* = 17.6, 10.2 Hz, 1H), 4.20 (t, *J* = 7.1 Hz, 1H), 3.44 (ddd, 1H), 3.29 (s, 1H).

### 3.2. Synthesis of 1-(1-methyl-1H-benzoimidazol-2-yl)-2-phenylethanamine (***2***)

Compound **1** (0.026 g, 0.056 mmol) was dissolved in 1 mL of dichloromethane, and then 200 µL of piperidine was added to the solution. The reaction was stirred at room temperature for 30 min, and then the solvent was removed by rotary evaporation and the obtained product used in the following step. Yield was quantitative. MS: + 252 (M+H^+^).

### 3.3. Synthesis of N,N′-(1,3-phenylenebis(methylene))bis(1-(1-methyl-1H-benzoimidazol-2-yl)-2-phenylethanamine) (***3***)

Compound **2** (0.014 g, 0.056 mmol) was dissolved in 1 mL of dichloromethane, and then 0.0037 g (0.028 mmol) of solid isophthaldialdehyde was added to the solution, which was subsequently stirred at room temperature overnight. The reaction was followed by mass spectrometry, and further compound **2** was added until the disappearance of the starting material. Sodium triacetoxyborohydride (STABH, 0.018 g, 0.084 mmol) was added to the solution, which was left under stirring for 3 h at room temperature. The reaction was stopped by removing the solvent by rotary evaporation, and then it was purified by column chromatography with silica 230–400 mesh as stationary phase and hexane:ethylacetate:methanol 85:15:20 as eluent. Yield was 69%. MS: +605 (M+H^+^).

### 3.4. Synthesis of N,N′-(1,3-phenylenebis(methylene))bis(1-(1-methyl-1H-benzoimidazol-2-yl)-N-((1-methyl-1H-imidazol-2-yl)methyl)-2-phenylethanamine) (***4***, mXPhI)

Compound **3** (0.080 g, 0.132 mmol) was dissolved in dichloroethane and brought to 0 °C. Then, 0.036 g of 1-methyl-2-imidazole carboxyaldehyde (0.331 mmol) was added to the solution, which was left under stirring for 30 min at this temperature. STABH (0.084 g, 0.397 mmol) was added, and the solution was left under stirring at a low temperature for 1 h. The reaction was followed by mass spectrometry. After 1 h, a further 0.331 mmol of 1-methyl-2-imidazole carboxyaldehyde was added, the reaction was stirred for 30 min, and then 0.084 g of STABH completed the reduction of the imine intermediate. The reaction was stopped by removing the solvent by rotary evaporation, and then the crude product was purified by column chromatography with silica 230–400 mesh as stationary phase and using hexane:ethyl acetate 9:1 + 1.5% of 28% aqueous ammonia solution as eluent. Yield was 8.3%. MS: +793 (M+H^+^), ^1^H NMR (400 MHz, MeOD): δ 7.76–7.63 (m, 2H), 7.39–7.19 (m, 7H), 7.11 (m, 13H), 6.88 (s, 2H), 6.83 (s, 2H), 4.33 (dd, *J* = 9.1, 5.4 Hz, 2H), 4.08 (d, *J* = 23.4 Hz, 2H), 3.99 (d, *J* = 13.8 Hz, 2H), 3.87 (d, *J* = 13.8 Hz, 2H), 3.83–3.69 (d, *J* = 23.4 Hz, 2H), 3.62–3.47 (m, 4H), 2.87 (s, 6H), 2.76 (s, 6H).

### 3.5. Synthesis of [Cu_2_(mXPhI)](ClO_4_)_2_

20 mg of ligand mXPhI were dissolved in methanol, and then 2 equiv. of Cu(ClO_4_)_2_.6H_2_O, dissolved in methanol, were added. After 15 min, the solution was poured into diethyl ether, and the copper complex precipitated as a brilliant blue solid. The solid was then filtered, washed three times with diethyl ether, and dried. Anal. Calcd. for C_50_H_52_Cl_4_Cu_2_N_10_O_16_.5H_2_O: C, 42.65; H, 4.44; N, 9.95. Found: C, 42.72; H, 4.58; N, 10.51. 

### 3.6. Spectrophotometric Titration of [Cu_2_(mXPhI)]^4+^ with NaN_3_

To a 5 × 10^−4^ M methanol solution of mXPhI were first added 2 equiv. of Cu(ClO_4_)_2_.6H_2_O, to generate in situ the dinuclear copper complex (initial volume: 2 mL). The resulting solution was titrated with a 4 × 10^−2^ M solution of sodium azide, until a ratio of 1:11 [Cu_2_(mXPhI)]^4^^+^:[N_3_^-^] was reached. Data were analyzed with a nonlinear least-square procedure [15], to extrapolate the binding constants for the 1:1 and 1:2 adducts.

### 3.7. Spectrophotometric Titration of [Cu_2_(mXPhI)]^4+^ with NaOH

To a 5 × 10^−4^ M methanol solution of mXPhI were first added 2 equiv. of Cu(ClO_4_)_2_.6H_2_O, to generate in situ the dinuclear copper complex (initial volume: 2 mL). The resulting solution was titrated with a 4 × 10^−2^ M solution of sodium hydroxide, until a ratio of 1:10 [Cu_2_(mXPhI)]^4^^+^:[OH^-^] was reached. Data were analyzed with a nonlinear least-square procedure [24], to obtain the species distribution plots. 

### 3.8. Oxidation of Chiral Catechols

The kinetics of oxidation of the chiral *ortho*-catechols L-Dopa, D-dopa, L-DopaOMe, D-DopaOMe, L-norepinephrine, and D-norepinephrine were studied by UV-vis spectroscopy, using a magnetically stirred and thermostated (at 25.0 ± 0.1 °C) optical cell of 1-cm path length. A mixture of acetate buffer (50 mM, pH 5.1) and methanol (1:10 *v*/*v*) was used as a solvent for the catalytic oxidations. The experiments were performed over the substrate concentration range between 5 × 10^−5^ and 1 × 10^−3^ M, while a constant concentration of 5 × 10^−6^ M of the copper complex was used. The experiments were initiated by adding to a solution of the substrate in the mixed aqueous/methanol buffer a few microliters of a methanol solution of the complex (final volume was 2.5 mL). The formation of the corresponding aminochrome was followed through the development of the band at 475 nm for L/D-Dopa and L/D-norepinephrine, and 468 nm for L/D-DopaOMe. In all the experiments, the noise was reduced by reading the absorbance difference between the λ_max_ of the aminochrome and that at 820 nm, and the initial rates of the oxidation were obtained by fitting the absorbance vs. time curves in the first few seconds of the reactions. To convert the rate data from Δabsorbance/time to M s^−1^, the absorbance changes were divided by the molar extinction coefficient of the aminochrome (3600 M^−1^cm^−1^) and the molar catalyst concentration. The dependence of the reaction rates of the catalytic reactions as a function of the substrate concentration exhibited, except for D-DopaOMe, hyperbolic behavior; therefore, the kinetic parameters (*k*_cat_, K_m_) were estimated, where possible, by fitting the data using a Michaelis–Menten equation. The enantio-differentiating capability in the catalytic reaction was evaluated through the two parameters R(*k*_cat_) and R(*k*_cat_/K_m_), defined above.

### 3.9. General Procedure for Asymmetric Sulfoxidation

To a solution of sulfide (10 mM) in 9:1 (*v*/*v*) methanol/acetate buffer 50 mM pH 5.1 (1 mL) was added [Cu_2_(mXPhI)]^4+^ (10 µM), and the mixture was stirred at room temperature. The reaction was initiated with addition of the appropriate volume of a solution of 0.3 M hydroxylamine, prepared in the same acetate buffer, and left to react overnight. For the determination of the reaction yield, a proper internal standard was added (final concentration: 0.2 mM), and the reaction mixture was analyzed by HPLC without any previous workup to prevent the loss of volatile reagent and product. Separation was operated with the Jasco HPLC and the reverse-phase column Ascentis express C18, 10 cm × 4.6 mm. The elution was carried out with flux of 0.8 mL/min by using 0.1% trifluoroacetic acid (TFA) in distilled water (solvent A) and 0.1% TFA in acetonitrile (solvent B).

For thioanisole, the elution started with a linear gradient from 5 to 100% B in 15 min and was followed by an isocratic elution for a further 5 min. The retention times were the following: methyl phenyl sulfoxide, 9.5 min; *p*-nitroacetophenone (as internal standard), 12.5 min; thioanisole, 14.2 min. 

For *p*-tolyl methyl sulfide, the elution started with 5 min 5% B and was followed by a linear gradient to 100% B in 7 min and by an isocratic elution for a further 8 min. The retention times were the following: methyl *p*-tolyl sulfoxide, 11.3 min; benzophenone (as internal standard), 13.6 min; *p*-tolyl methyl sulfide, 14.1 min. 

### 3.10. Determination of Enantiomeric Excess

The sulfide oxidations were carried out as before, but prior to HPLC analysis the reactions were quenched with the addition of 1 M HClO_4_. Methanol was removed by rotary evaporation, and the remaining aqueous phase was extracted with three portions of dichloromethane; then, the collected extracts were dried over sodium sulfate, filtered, and evaporated to give the crude product for HPLC analysis. The determination of enantiomeric excess was performed with the Shimadzu HPLC equipped with the direct-phase column Lux 5u amylose-2 250 × 4.60 mm and using *n*-hexane (solvent A) and 2-propanol (solvent B) at 30 °C. With thioanisole, the elution, at 0.9 mL/min, started with 5 min 5% B and was followed by a linear gradient to 15% B in 35 min and by an isocratic elution for a further 10 min. The retention times were 37.2 min for the *(S)*-methyl phenyl sulfoxide isomer and 38.0 min for the *R* isomer. With methyl *p*-tolyl sulfide, the elution, at 0.8 mL/min, was performed in isocratic mode with 10% B. The retention times were 15.6 min for the *(S)*-methyl tolyl sulfoxide isomer and 17.2 min for the *R* isomer. 

The attribution of the peaks in the HPLC runs to the *R* or *S* enantiomers of the sulfoxide was obtained by preparing S-methyl phenyl sulfoxide and *(S)*-methyl tolyl sulfoxide with an excess of the *S* isomer by means of the enzyme tyrosinase, according to a published method [21].

### 3.11. Experiment with 18-O_2_

A solution of thioanisole (10 mM) in 9:1 (*v*/*v*) methanol/acetate buffer 50 mM pH 5.1 (1 mL) and [Cu_2_(mXPhI)]^4+^ (10 µM) was purged for 20 min with nitrogen to obtain anaerobic conditions. The reaction was initiated with the addition of hydroxylamine (final concentration: 0.5 mM) and then ^18^O_2_ added with a gas-tight syringe and was then left to react overnight. The reaction mixture was analyzed by ESI-MS/MS without any previous workup. The similarity in fragmentation pattern of the commercial methyl phenyl sulfoxide and the 18-O analogue enabled us to confirm the same structure for these two products (Figure S5a,b). From the enlargement of the ESI-MS spectrum of methyl phenyl sulfoxide (Figure S5c), it was possible to determine the percentage of incorporation of 18-O, which was estimated as 77%.

## 4. Conclusions

Through the synthesis of the ligand mXPhI, we developed a new versatile synthetic pathway for obtaining biomimetic complexes related to [Cu_2_(L55)]^4+^ and exploiting the backbone of natural amino acids (L-phenylalanine in the present case) to introduce chirality into the ligand. As with previously reported dinuclear complexes of the same family [10,11,12,19], the oxidation of biogenic catechols disclosed significant chiral discrimination only for the couple L/D-Dopa methyl ester, probably due to the different approach to the catalyst active center, as recently discussed [9]. An important advancement is made here in the asymmetric sulfoxidation of organic sulfides. For the first time, in fact, the catalytic monooxygenase reaction promoted by a biomimetic dinuclear copper(II) complex yields sulfoxide with significant e.e. The improvement in enantioselectivity is totally due to bringing the chiral center of the ligand into the immediate coordination sphere of the complex, whereas for the parent [Cu_2_(L55Bu_4_)]^4+^ complex the chiral substituents were directed outside the coordination sphere [10]. Although the subsequently reported [Cu_2_(EHI)]^4+^ [11] and [Cu_2_(mXHI)]^4+^ [12] complexes bore chiral centers within histidine chelate rings, the ligands were devoid of the benzimidazole rings which appear to be critical for the effective interaction with hydrophobic substrates such as the alkyl aryl sulfides. Other types of stereoselective oxidations promoted by [Cu_2_(mXPhI)]^4+^ are currently under scrutiny in our laboratory.

## Figures and Tables

**Table 1:** Catalytic oxidation of thioanisole and p-tolyl methyl sulfide (10 mM) by [Cu_2_(mXPhI)]^4+^ (10 µM) in methanol–acetate buffer (50 mM, pH 5.1) 9:1 (*v*/*v*), at various concentrations of hydroxylamine. Reaction yields are calculated with respect to the reducing agent, and enantiomeric excess refers to the product sulfoxides.

[NH_2_OH], mM	Substrate	Yield %	e.e. %	TON ^a^
0.3	thioanisole	35%	18%	10.5
0.5	thioanisole	13%	32%	6.5
1	thioanisole	6%	43%	6
5	thioanisole	0.5%	36%	2.5
10	thioanisole	trace	19%	trace
0.3	*p*-tolyl methyl sulfide	ND ^b^	~40%	ND
0.5	*p*-tolyl methyl sulfide	6	39%	3
1	*p*-tolyl methyl sulfide	3	38%	3
5	*p*-tolyl methyl sulfide	0.3	27%	1.5
10	*p*-tolyl methyl sulfide	trace	racemic	trace

^a^ TON: turnover number. ^b^ ND: not determinable due to the low area of the HPLC peak.

**Table 2:** Kinetic parameters for oxidation of chiral catechols in methanol/50 mM acetate buffer, pH = 5.1, 10:1 *v/v*, at 25 °C.

Substrate	K_M_ (mM)	*k*_cat_ (s^−1^)	*k*_cat_/K_M_ (M^−1^ s^−1^)	R(*k*_cat_) %	R(*k*_cat_/K_M_) %
L-Dopa	(7.37 ± 0.16) × 10^−2^	(2.20 ± 0.13) × 10^−3^	29.9	3%	−3%
D-Dopa	(6.52 ± 0.89) × 10^−2^	(2.14 ± 0.07) × 10^−3^	32.8		
L-DopaOMe	(1.35 ± 0.14) × 10^−1^	(3.93 ± 0.13) × 10^−2^	29.1		
D-DopaOMe	-	-	-		
R-(-)-norepinephrine	(8.26 ± 0.52) × 10^−2^	(2.29 ± 0.04) × 10^−3^	27.7	3%	−10%
S-(+)-norepinephrine	(6.38 ± 0.41) × 10^−2^	(2.17 ± 0.04) × 10^−3^	34.0		

## Data Availability

Data is contained within the article or supplementary material.

## Supplementary Materials

The following supporting information can be downloaded at: https://www.mdpi.com/article/10.3390/molecules28114487/s1: structure of the active site of met tyrosinase (Figure S1), additional UV-vis spectra (Figure S2), reaction yields (Figures S3 and S4), mass spectra (Figure S5), kinetic traces (Figure S6), and derivation of the kinetic equation.
