# Supplementary material for: Asymmetric Sulfoxidation by a Tyrosinase Biomimetic Dicopper Complex with a Benzimidazolyl Derivative of L-Phenylalanine
Source: Molecules. 2023 Jun 1;28(11):4487. doi: 10.3390/molecules28114487 (PMC10254291; doi:10.3390/molecules28114487)
Supplement: Supplementary file 1 [file molecules-28-04487-s001.zip › molecules-2395663-supplementary.pdf]

Supporting information

Asymmetric sulfoxidation by a tyrosinase biomimetic dicopper complex with a benzimidazolyl derivative of L-phenylalanine

Eliana Lo Presti,[1] Fabio Schifano,[1] Chiara Bacchella,[1] Laura Santagostini,[2] Luigi Casella[1] and Enrico Monzani[1*]

[1] Dipartimento di Chimica, Università di Pavia, Via Taramelli 12, 27100 Pavia, Italy
[2] Dipartimento di Chimica, Università di Milano, Via Golgi 19, 20133 Milano, Italy

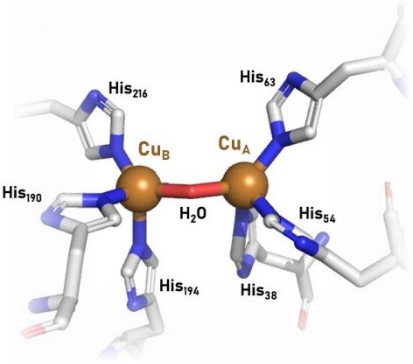

Figure S1. X-ray crystal structure of the active site of the Met1-form (one water molecule) of *S. castaneo globisporus* tyrosinase (this tyrosinase does not have the His-Cys crosslink present in other tyrosinases).

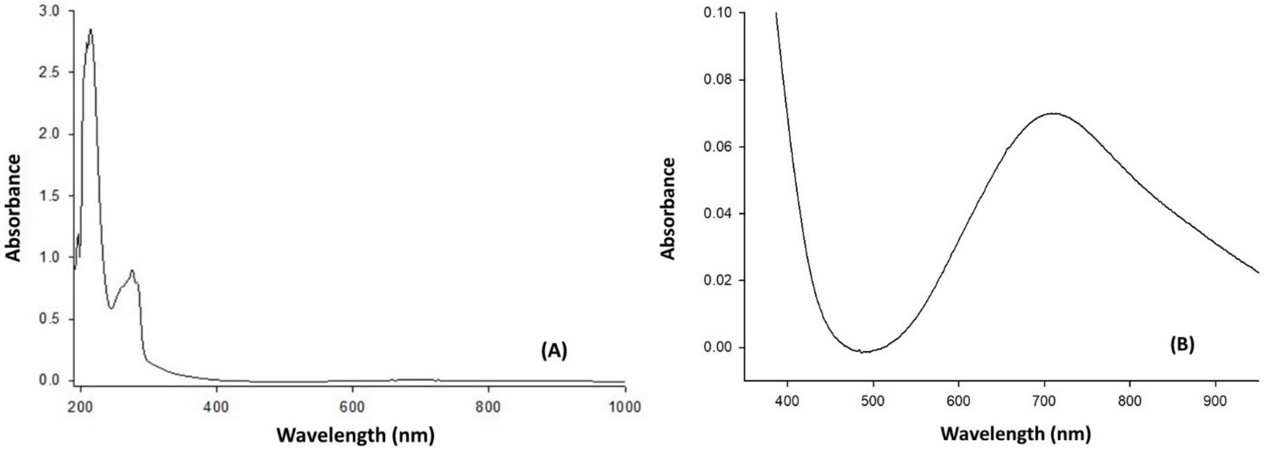

Figure S2. UV-Vis electronic spectrum of $[Cu_2(mXPhI)]^{4+}$ in methanol solution: (A) in the UV range 0.1 mM concentration, and (B) in the Visible region 0.5 mM concentration of the complex.

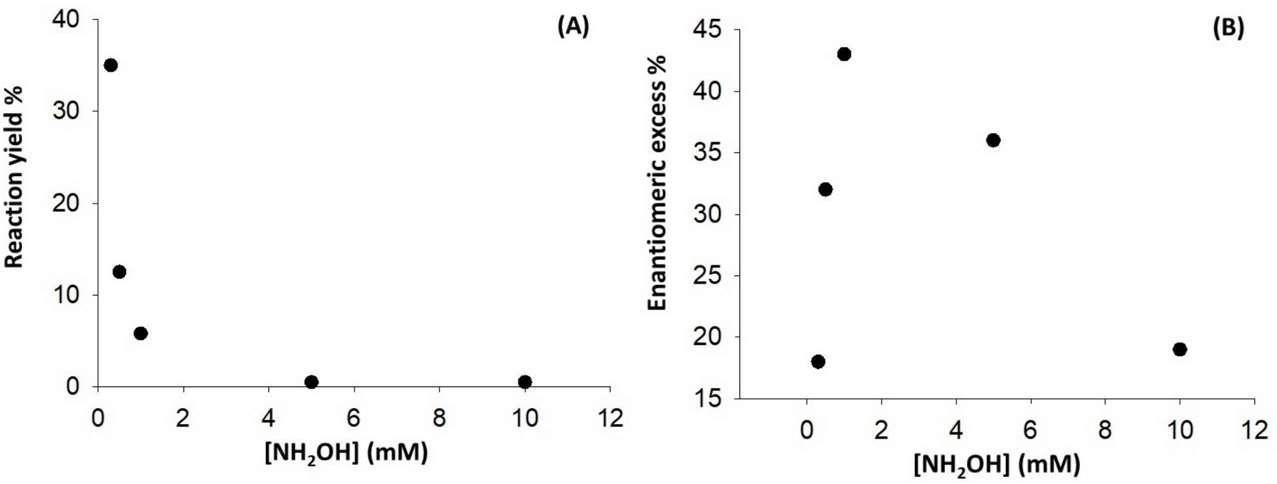

Figure S3. Reaction yield (A) and enantiomeric excess trend (B) in oxidation of thioanisole promoted by $[Cu_2(mXPhI)]^{4+}$

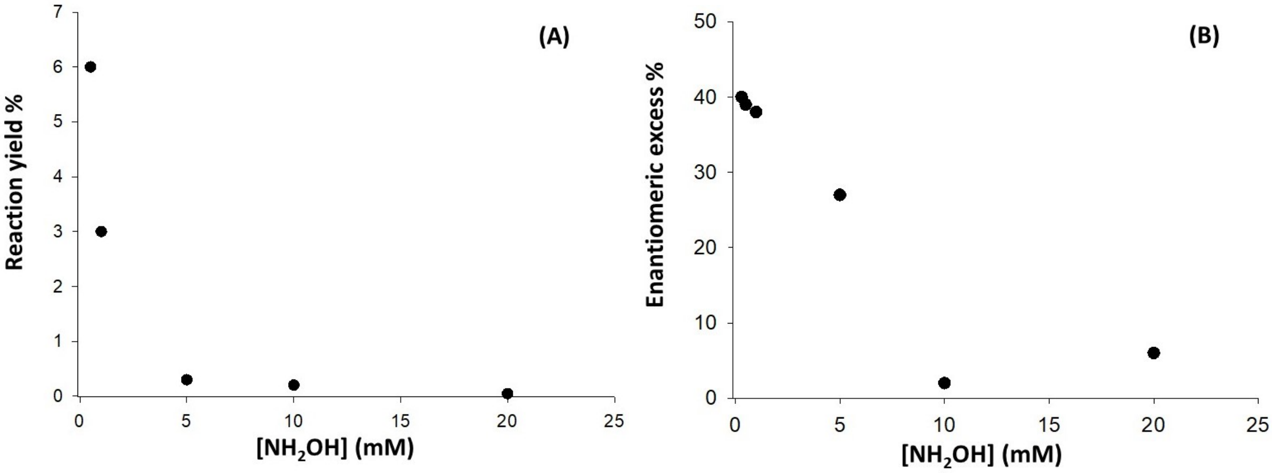

Figure S4. Reaction yield (A) and enantiomeric excess trend (B) in oxidation of methyl *p*-tolyl sulfide promoted by $[Cu_2(mXPhI)]^{4+}$

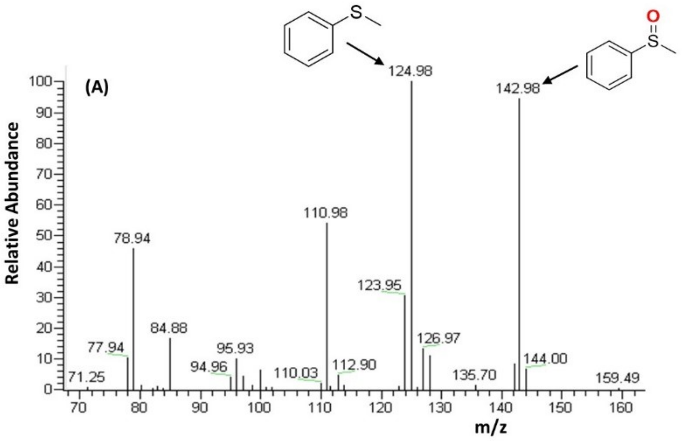

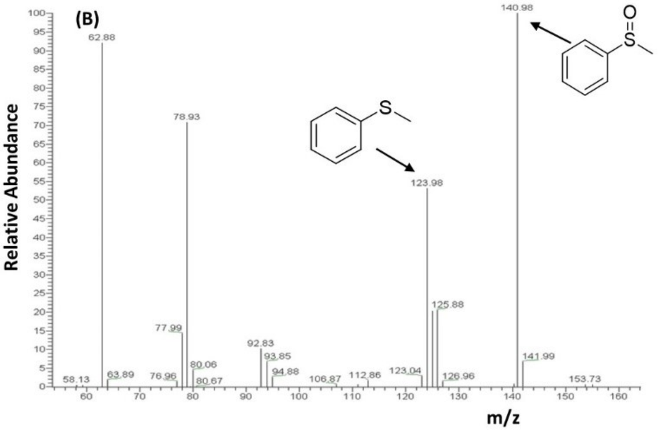

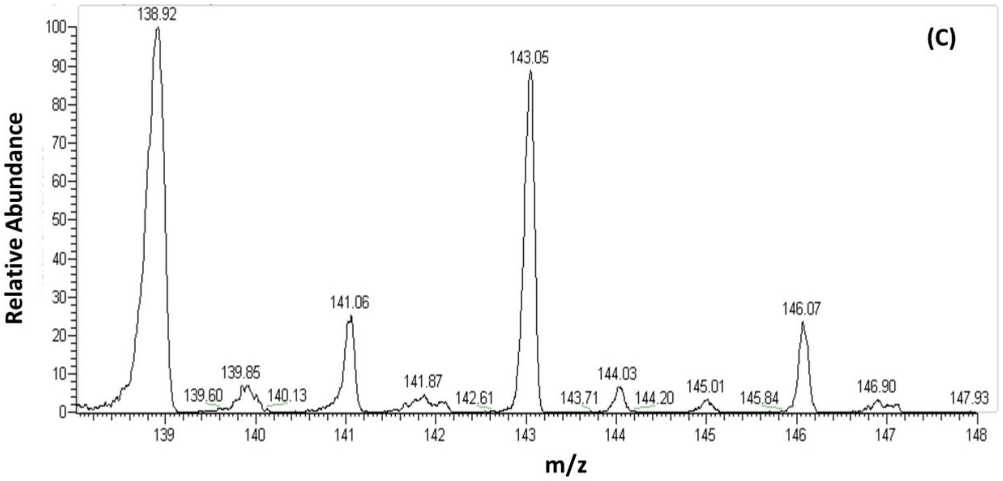

Figure S5. Sulfoxidation of thioanisole in presence of oxygen-18; (A) Fragmentation pattern of the methylphenyl sulfoxide including O-18 (18-O-methylphenyl sulfoxide: m/z = +143); (B) Fragmentation pattern of commercial methylphenyl sulfoxide (methylphenyl sulfoxide: m/z = +141); (C) Enlargement of the ESI-MS spectrum.

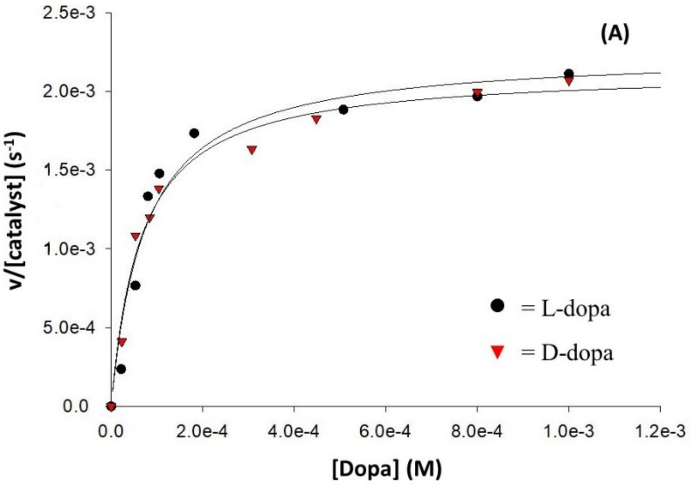

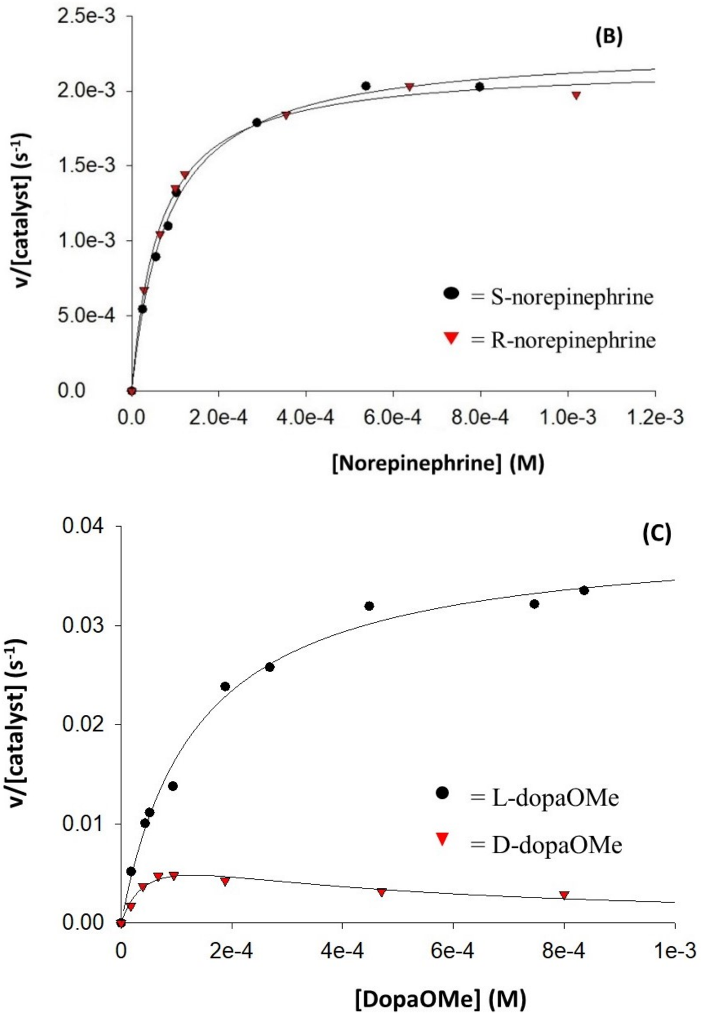

Figure S6. Effect of substrate concentration on initial rate of oxidation of A) L-/D-Dopa, B) R-/S-norepinephrine, C) L-/D-DopaOMe promoted by $[Cu_2(mXPhI)]^{4+}$ 5 μM, in 10:1 MeOH:acetate buffer (50 mM, pH = 5.1), temperature: 25 °C.

Derivation of the kinetic equation to describe D-Dopa methyl ester inhibition effect:

The kinetic equation describing the biphasic behavior observed in the catecholase activity of $[Cu_2(mXPhI)]^{4+}$ (here indicated as C) toward D-Dopa (indicated as S) can be obtained considering that the complex binds two substrate molecules in a stepwise process forming CS (the copper complex bound by one S molecule) and CS_2 (the complex bound by two S molecules). Both CS and CS_2 are active in the product (P) formation but with different rate constants.

Assuming the following equilibria:

$C + S \rightleftarrows CS$, ruled by K_{B1}

$CS + S \rightleftarrows CS_2$, ruled by K_{B2}

and

$CS \longrightarrow P$, ruled by k_{c1}

$CS_2 \longrightarrow P$, ruled by k_{c2}

Indicating with $[C_0]$ the initial $[Cu_2(mXPhI)]^{4+}$ concentration in the catalysis, the mass balance leads to the following equation:

$[C_0] = [C] + [CS] + [CS_2]$

appropriate substitutions give

$[C_0] = [C] + K_{B1} \times [C] \times [S] + K_{B2} \times [CS] \times [S] = [C] + K_{B1} \times [C] \times [S] + K_{B1} \times K_{B2} \times [C] \times [S]^2$

so

$$[C] = \frac{[C_0]}{1+K_{B1}\times[S]+K_{B1}\times K_{B2}\times[S]^2} \quad ; \quad [CS] = \frac{K_{B1}\times[S]\times[C_0]}{1+K_{B1}\times[S]+K_{B1}\times K_{B2}\times[S]^2} \quad ; [CS_2] = \frac{K_{B1}\times K_{B2}\times[C_0]\times[S]^2}{1+K_{B1}x[S]+K_{B1}\,x\,K_{B2}[S]^2}$$

The substitution of [CS] and $[CS_2]$ in the rate kinetic equation ($r = k_{c1} \times [CS] + k_{c2} \times [CS_2]$) leads to:

$$r = \frac{k_{c1}\times K_{B1}\times[S]\times[C_0]+k_{c2}\times K_{B1}\,x\,K_{B2}\times[C_0]\times[S]^2}{1+K_{B1}x[S]+K_{B1}xK_{B2}[S]^2}$$

Dividing for $[C_0]$ and K_{B1} we obtained the final equation (where $K_M = 1/K_{B1}$):

$$r/[C_0] = \frac{k_{c1}\times[S]+k_{c2}\times K_{B2}\times[C_0]\times[S]^2}{K_M+[S]+K_{B2}\times[S]^2}$$
